# Regulation and Functions of 15-Lipoxygenases in Human Macrophages

**DOI:** 10.3389/fphar.2019.00719

**Published:** 2019-07-04

**Authors:** Ryan G. Snodgrass, Bernhard Brüne

**Affiliations:** Faculty of Medicine, Institute of Biochemistry I, Goethe-University Frankfurt, Frankfurt, Germany

**Keywords:** lipoxygenase, macrophage, lipid mediator, inflammation, immunity, cholesterol

## Abstract

Lipoxygenases (LOXs) catalyze the stereo-specific peroxidation of polyunsaturated fatty acids (PUFAs) to their corresponding hydroperoxy derivatives. Human macrophages express two arachidonic acid (AA) 15-lipoxygenating enzymes classified as ALOX15 and ALOX15B. ALOX15, which was first described in 1975, has been extensively characterized and its biological functions have been investigated in a number of cellular systems and animal models. In macrophages, ALOX15 functions to generate specific phospholipid (PL) oxidation products crucial for orchestrating the nonimmunogenic removal of apoptotic cells (ACs) as well as synthesizing precursor lipids required for production of specialized pro-resolving mediators (SPMs) that facilitate inflammation resolution. The discovery of ALOX15B in 1997 was followed by comprehensive analyses of its structural properties and reaction specificities with PUFA substrates. Although its enzymatic properties are well described, the biological functions of ALOX15B are not fully understood. In contrast to ALOX15 whose expression in human monocyte-derived macrophages is strictly dependent on Th2 cytokines IL-4 and IL-13, ALOX15B is constitutively expressed. This review aims to summarize the current knowledge on the regulation and functions of ALOX15 and ALOX15B in human macrophages.

## Introduction

Lipoxygenases (LOXs) are non-heme iron-containing dioxygenases that catalyze the stereo-specific peroxidation of polyunsaturated fatty acids (PUFAs) containing one or more 1,4-*cis*,*cis* pentadiene moieties to the corresponding hydroperoxy derivatives (Kuhn et al., 2018). In mammals, LOX enzymes are expressed in numerous cell types including epithelial, endothelial, and immune cells and are involved in various functions including skin barrier formation, cell differentiation, and immunity (Kuhn et al., 2015). The human genome contains six functional LOX genes (*ALOX15*, *ALOX15B*, *ALOX12*, *ALOX12B*, *ALOX5*, and *ALOXE3*) each encoding a distinct LOX enzyme (Ivanov et al., 2015). All mammalian LOXs are single polypeptide chain proteins that fold into a two-domain structure (Kuhn et al., 2015). The smaller N-terminal domain consists of several parallel and anti-parallel β-sheets that regulate activity and facilitate membrane binding. The C-terminal catalytic domain consists of several helices and contains the catalytic non-heme iron localized in the putative substrate-binding pocket.

Macrophages are versatile immune cells strategically positioned throughout body tissues (Varol et al., 2015). They are endowed with a broad functional repertoire of sensors allowing them to respond to a variety of environmental cues and acquire diverse but specialized functional phenotypes crucial for orchestrating initiation, progression, and the resolution of inflammation (Murray et al., 2014). In addition to classically activated pro-inflammatory macrophages and anti-inflammatory macrophages, resolution-phase macrophages are immune regulatory, endowed with aspects of both pro-inflammatory and anti-inflammatory macrophages (Stables et al., 2011). Resolution-phase macrophages are highlighted by the strong up-regulation of arachidonate 15-lipoxygenase (ALOX15), a key enzyme involved in the synthesis of specialized pro-resolving mediators (SPMs) including lipoxins (LXs), resolvins (Rvs), protectins, and maresins that facilitate inflammation resolution (Buckley et al., 2014). For this reason, ALOX15 has attracted much attention for its role in contributing to active resolution of the inflammatory process. Interestingly enough, ALOX15 is not an exclusive 15-lipoxygenating enzyme. Arachidonate 15-lipoxygenase type B (ALOX15B), which is also expressed in human macrophages (Wuest et al., 2012), catalyzes the stereo-specific peroxidation of PUFAs to the same hydroperoxy derivatives as ALOX15 (Kutzner et al., 2017). Furthermore, in contrast to ALOX15 whose expression is restricted to certain macrophage phenotypes, ALOX15B is constitutively expressed in human macrophages (Wuest et al., 2012; Snodgrass et al., 2018). Knockout experiments suggest that the various mammalian LOX enzymes exhibit different biological functions. In this respect, the multiplicity of 15-lipoxygenase enzymes in human macrophages likely does not reflect functional redundancy but rather specialized biological functions. This review aims to summarize our cumulative understanding of the roles of ALOX15 and ALOX15B in human macrophages.

## Activity of Human ALOX15 and ALOX15B Enzymes

LOXs oxygenate PUFAs including linoleic acid (LA; C18:Δ2, ω-6), alpha-linolenic acid (ALA; C18:Δ3, ω-3), gamma-linolenic acid (GLA; C18:Δ3, ω-6), eicosapentaenoic acid (EPA; C20:Δ5, ω-3), and docosahexaenoic acid (DHA; C22:Δ6, ω-3) to their corresponding hydroperoxy derivative but were traditionally classified with respect to their positional specificity of arachidonic acid (AA; C20:Δ4, ω-6) oxygenation (Kuhn et al., 2015). Following the discovery of its rabbit ortholog in immature red blood cells (Schewe et al., 1975), human ALOX15 was reported to oxygenate AA primarily at carbon 15 producing 15-hydroxyeicosatetraenoic (HETE), hence its name (Sigal et al., 1990). Subsequent studies lead to the identification of a second human LOX capable of oxygenating AA at carbon 15, which was given the name ALOX15B (Brash et al., 1997). To differentiate between genes and proteins in this review, we use traditional formatting conventions in which gene symbols are italicized while symbols for proteins are not italicized. Symbols composed of uppercase letters refer to human genes and proteins while lowercase letters refer to non-human and murine genes and proteins. Although LOX nomenclature appears straightforward, the classification system fails to take into consideration the extent of each enzyme’s reaction specificity, which can lead to confusion and misunderstanding (**Table 1**).

**Table 1:** Human 15-lipoxygenase genes and their murine orthologs.

	Gene symbol	Gene name	Commonly used synonyms
Human	*ALOX15*	Arachidonate 15-lipoxygenase	*15-LOX, 15-LOX-1, 12/15-LOX, 15-LO*
Mouse	*Alox15*	Arachidonate 15-lipoxygenase	*15-LOX, 12/15-LO, 12/15-LOX, 15-LO*
Human	*ALOX15B*	Arachidonate 15-lipoxygenase type B	*15-LOX-2, 15-LOX2, 15-LOX-B*
Mouse	*Alox8*	Arachidonate 8-lipoxygenase	*Alox15b, 8-LOX*

For example, oxygenation of AA by human ALOX15 and orthologs of higher primates including chimpanzees produce predominately 15-HETE and small amounts of 12-HETE while murine Alox15 and orthologs from mammals ranked lower in evolution, including rats and pigs, produce only small amounts of 15-HETE but primarily 12-HETE (Adel et al., 2016; Kuhn et al., 2018). In contrast to ALOX15, ALOX15B exclusively produces 15-HETE while its murine ortholog Alox15b (also named Alox8 which is encoded by the *Alox8* gene) is an 8-lipoxygenating enzyme producing 8-HETE from AA substrate (Ivanov et al., 2015). Furthermore, the reaction specificities of 15-LOXs with omega-3 PUFAs are variable and cannot be predicted or inferred from the product pattern of AA oxygenation (Kutzner et al., 2017). With increasing diversity of the LOX family, a sequence-related classification system based on enzyme characteristics including the degree of amino acid sequence conservation, genomic organization, catalytic similarity, and evolutionary relatedness rather than the traditional AA specificity-based classification system has been suggested (Ivanov et al., 2015).

**Figure 1:**
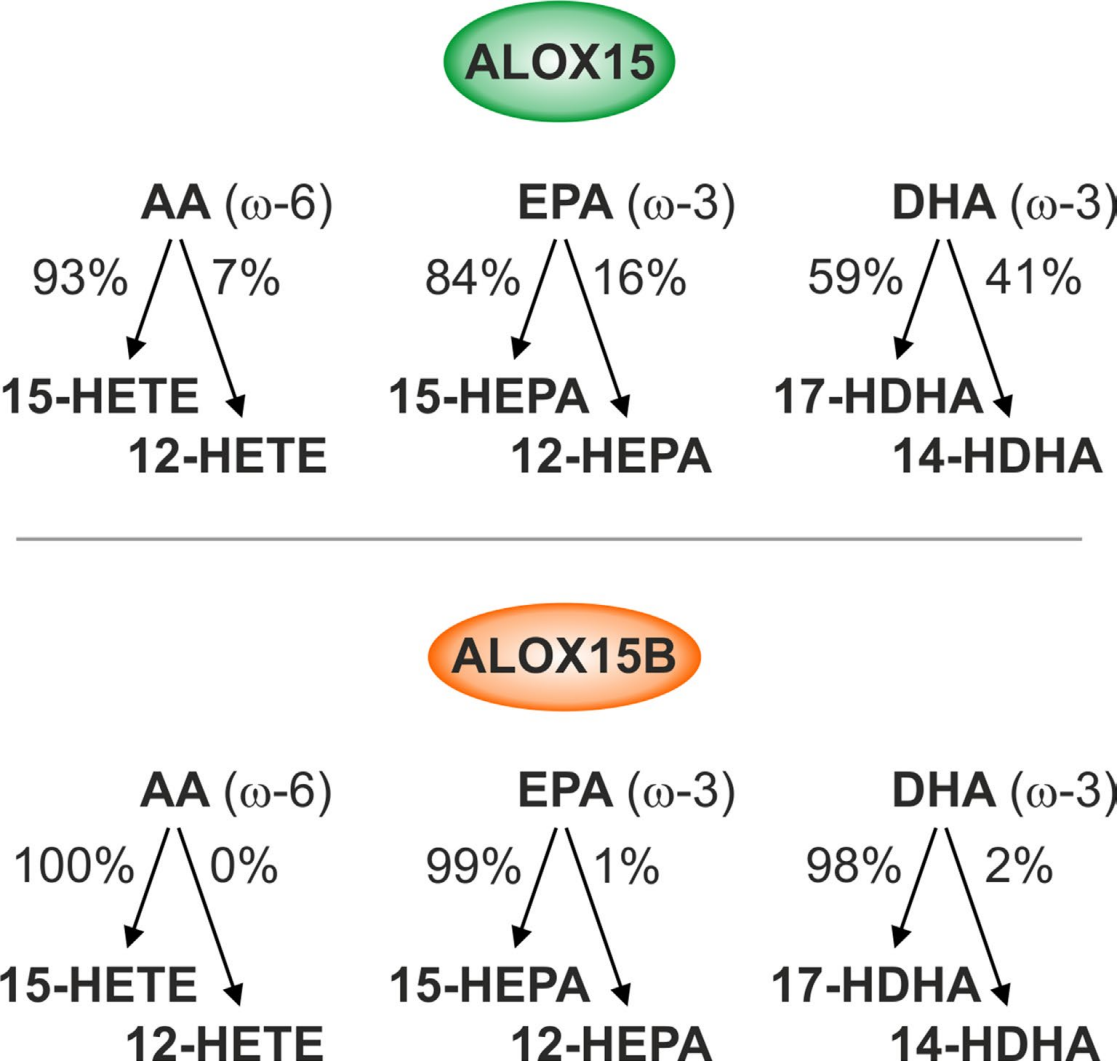
Reaction specificities of human ALOX15 and ALOX15B. Enzymatic activity of recombinant human ALOX15 and ALOX15B with ω-6 and ω-3 free polyunsaturated fatty acid (PUFA) substrates. ALOX15 exhibits dual reaction specificity while ALOX15B exhibits singular reaction specificity of arachidonic acid (AA), eicosapentaenoic acid (EPA), and docosahexaenoic acid (DHA) oxygenation.

Although SPMs are widely recognized for their role in resolving inflammation and stimulating tissue regeneration, the monohydroxy fatty acid products of the 15-lipoxygenase reaction also exhibit biological activity and are thought to serve various physiological functions (Ivanov et al., 2015). AA-derived 15- and 12-HETE exhibit both pro- and anti-inflammatory effects (Singh and Rao, 2019). 15-HETE has been reported to bind and activate peroxisome proliferator-activated receptor γ (PPARγ) in both human and murine macrophages (Huang et al., 1999) while 12-HETE was shown to activate extracellular signal-regulated kinase 1/2 (ERK1/2) and nuclear factor kappa-light-chain-enhancer of activated B cells (NF-κB) *via* G protein-coupled receptor 31 (GPR31) (Guo et al., 2011). Human macrophages incubated with 12- and 15-HETE showed increases in LPS-induced gene expression (Namgaladze et al., 2015) while ALOX15 was reported to induce the production of pro-inflammatory cytokine IL-12 in macrophages (Li et al., 2013). LA-derived 13-hydroxyoctadecadienoic acid (HODE) was shown to both activate PPARγ and suppress PPARδ activity (Huang et al., 1999; Shureiqi et al., 2003; Zuo et al., 2006) as well as induce oxidative stress, ER stress, and apoptosis in murine hepatoma cells (Zhang et al., 2017). Although 15- and 12-hydroxyeicosapentaenoic acid (HEPA) derived from EPA and 17- and 14-hydroxy-DHA (HDHA) generated from DHA function as intermediate precursors for the biosynthesis of Rvs, protectins, and maresins (Buckley et al., 2014), biological functions of these individual omega-3 monohydroxy fatty acids remain limited. Recently, insight into the monohydroxylated fatty acid product patterns catalyzed by human ALOX15 and ALOX15B was investigated using recombinant enzyme preparations with different substrate PUFAs (**Figure 1**) (Kutzner et al., 2017).

Omega-6 PUFAs are the most abundant polyenoic fatty acids in mammalian cells and are therefore major lipoxygenase (LOX) substrates. Using AA as substrate, ALOX15 displayed dual reaction specificity forming both 15-HETE and 12-HETE in a 9:1 ratio, confirming original experiments performed with its purified rabbit ortholog (Bryant et al., 1982). In contrast to ALOX15, ALOX15B exhibited singular positional specificity producing only 15-HETE. When LA was used as substrate, 13-HODE was the dominant product for both ALOX15 and ALOX15B. The omega-3 PUFAs, including EPA and DHA, are also present in mammalian cells but at much lower concentrations than omega-6 PUFAs (Calder, 2008). When EPA was employed as substrate, ALOX15 showed a more pronounced dual positional specificity compared to AA producing both 15-HEPA and 12-HEPA in an 8.5:1.5 ratio while ALOX15B exhibited singular positional specificity producing only 15-HEPA. With DHA as substrate, ALOX15-catalyzed oxygenation produced nearly equal amounts of 17-HDHA and 14-HDHA. In contrast, ALOX15B produced 17-HDHA almost exclusively (Kutzner et al., 2017). *In vitro*, both ALOX15 and ALOX15B produce identical oxygenation products, albeit at different ratios reflecting their singular and dual specificity to free PUFA substrates. The biological implication of these differences is currently unknown; however, the dual reaction specificity unique to ALOX15 that generates 12-HETE, 12-HEPA, and 14-HDHA might be advantageous for SPM biosynthesis in macrophages.

Unlike other mammalian LOX isoforms, both ALOX15 and ALOX15B are unique in their ability to oxygenate esterified PUFAs found in biomembranes, lipoproteins, and cholesteryl esters (CEs) in addition to free fatty acid substrates (Schewe et al., 1975; Belkner et al., 2000; Hammond and O’Donnell, 2012; Hutchins and Murphy, 2012; Bender et al., 2016; Wenzel et al., 2017). In 2007, analysis of ionophore-activated IL-4-treated human monocytes revealed four esterified 15-HETEs, subsequently identified as 3 plasmalogen and 1 acyl phosphatidylethanolamine [18:0p, 18:1p, 16:0p, and 18:0a/15-HETE-phosphatidylethanolamine (PE)] (Maskrey et al., 2007). Further analysis revealed that these products were generated by ALOX15 through direct oxidation of the intact phospholipid (PL) and comprised approximately 30% of the total 15-HETE generated (O’Donnell and Murphy, 2012). Within the monocyte PL fractions, over 90% of 15-HETE was found incorporated in PE, while approximately 1.5% of the total cellular PE pool was found to contain 15-HETE (O’Donnell and Murphy, 2012). Both human ALOX15B and its murine ortholog Alox15b have also been shown to oxidize esterified PUFAs in solubilized PLs as well as in bilayer PLs encompassed in nanodiscs (Coffa and Brash, 2004; Bender et al., 2016). Following incubation of ALOX15B with PL-esterified-AA containing nanodiscs, 15-HETE but not 12-HETE was formed as product corroborating the *in vitro* data, which demonstrated the singular positional specificity of ALOX15B with AA substrate (Kutzner et al., 2017). Moreover, when exogenous ALOX15B was added to crude HEK cell lysates, 15-HETE and LA oxygenation product 13-HODE were only detected following treatment with phospholipase A_2_, likely indicating that the products were generated as the esterified substrate. A key difference between 15-LOX-mediated oxygenated PLs and oxygenated free PUFAs is that oxygenated PLs do not get secreted but remain cell associated, residing within membranes. Although unlikely to mediate high-affinity receptor–ligand interactions, oxidized PLs (oxPLs) likely exert their effects through low-affinity interactions with proteins and by altering membrane electronegativity, which influences protein–membrane interactions (O’Donnell et al., 2019). Emerging evidence indicates that, enzymatically, oxPLs have profound biological activity in macrophages including blocking cell surface and soluble pattern recognition receptors including TLR4, CD14, and LPS-binding protein as well as orchestrating the nonimmunogenic clearance of apoptotic cell (AC)-derived autoantigens and maintaining self-tolerance during inflammation (Uderhardt et al., 2012; Rothe et al., 2015). In non-macrophage immune cells including eosinophils and platelets, ALOX15-derived HETE-PLs enhance the ability of phosphatidylserine to interact with multiple clotting factors, increasing the rates of thrombin generation that facilitate hemostasis (Uderhardt et al., 2017; O’Donnell et al., 2019).

Intracellular cholesterol can be esterified with fatty acids to form CEs, which have been shown to be substrates for 15-lipoxygenases (Belkner et al., 1993; Hutchins et al., 2011; Hutchins and Murphy, 2012). Because the oxidation of CEs, which are a major component of low-density lipoproteins (LDLs), is frequently cited in the transformation of macrophages to foam cells during progression of atherosclerotic lesions, the role of 15-lipoxygenases in oxidized CE formation has been investigated. Human atherosclerotic lesions contain large amounts of oxygenated PUFAs with derivatives of LA being the most predominant (Rapp et al., 1983; Kühn et al., 1992). Of the oxygenated PUFAs in lesions, more than 85% are localized in CEs (Kühn et al., 1992). Furthermore, it was reported that 23% of cholesteryl linoleate (18:2-CE), 16% of cholesteryl arachidonate (20:4-CE), and 12% of cholesteryl docosahexaenoate (22:6-CE) in human atherosclerotic plaques became oxidized through enzymatic and non-enzymatic processes (Hutchins et al., 2011). To gain insight into the role of 15-lipoxygenase in mediating oxidized CE (oxCE) generation in macrophages, Hutchins et al. measured the oxCEs in wild-type and Alox15-deficient murine resident peritoneal macrophages following incubation with human lipoprotein CEs (Hutchins and Murphy, 2012). Alox15-specific oxidation products of 18:2-CE and 20:4-CE were consistently present in wild-type cells but could not be detected in macrophages lacking Alox15. Further examination of the metabolic fate of Alox15-mediated oxCEs revealed a robust intracellular remodeling pathway whereby hydrolysis of the oxidized fatty acyl chain and subsequent reacylation generated oxidized phosphatidylcholines (PCs) (Hutchins and Murphy, 2012). These results demonstrate that oxCE remodeling contributes to PC oxidation and suggests that the presence of abundant oxCE may influence overall PC oxidation levels in macrophages. Moreover, oxidation of the acyl chain of the CEs has been suggested to enhance the propensity for hydrolysis as oxidized 18:2-CE was shown to be preferentially hydrolyzed over its non-oxidized counterpart by macrophage CE hydrolases at both neutral and acidic pH (Belkner et al., 2000). Collectively, these works suggest that by oxidizing intracellular CEs, 15-lipoxygenases may facilitate the hydrolysis and subsequent mobilization of oxidized acyl species for incorporation into various cellular compartments.

Upon cellular stimulation with calcium ionophore, both ALOX15 and ALOX15B increase localization at plasma membrane and at the cytoplasmic side of intracellular membranes (Brinckmann et al., 1998; Bender et al., 2016). Insight into how ALOX15 and ALOX15B undergo localization to biological membranes and selectively oxygenate individual PUFA-PL substrates from the diversified PLs was recently investigated. Using various ALOX15 and ALOX15B-expressing cell types, Wenzel et al. reported that a promiscuous small scaffolding protein, phosphtidylethanolamine-binding protein 1 (PEBP1), forms complexes with ALOX15 and ALOX15B, which can be further increased following stimulation with IL-13 and lipopolysaccharide (LPS), respectively (Wenzel et al., 2017). In the absence of PEBP1, both ALOX15 and ALOX15B exert high enzymatic oxidation of free AA but low activity towards esterified AA-PE. Upon addition of PEBP1, the oxidation of AA-PE by both LOX isoforms was markedly increased. Mechanistic insight showed that PEBP1 contains multiple sites for binding free AA and through binding reduces AA levels accessible for oxygenation. By “depleting” endogenous AA and complexing with ALOX15 or ALOX15B, PEBP1 redirects AA-PE as enzymatic substrate for 15-LOXs. Whether PEBP1 binds other PUFAs in addition to AA to facilitate oxygenation of the corresponding PUFA-PLs was not reported.

## Regulation of ALOX15 and ALOX15B Expression

**Figure 2:**
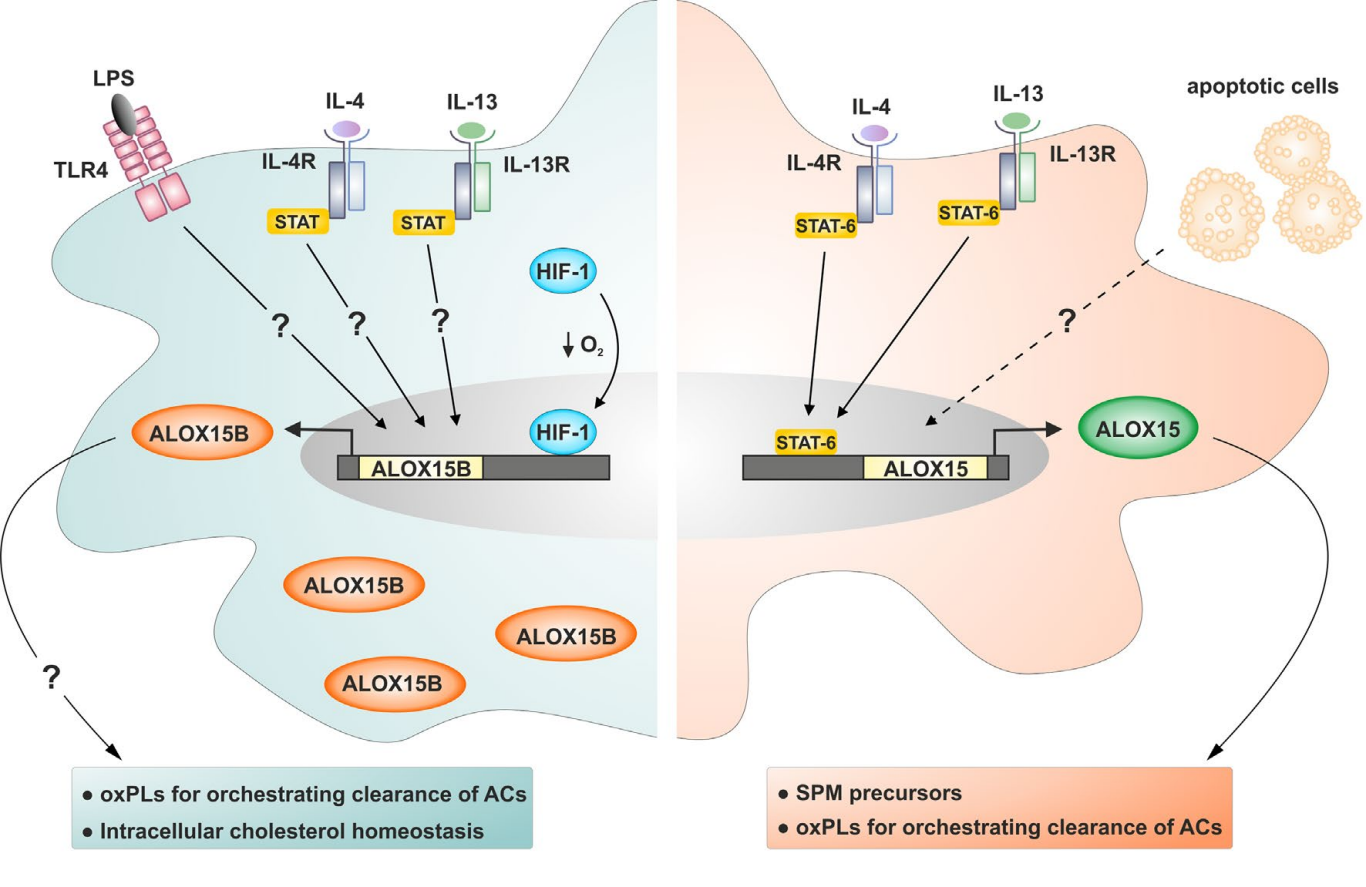
Regulation of ALOX15B and ALOX15 expression in human macrophages. ALOX15B protein is constitutively present in human monocyte-derived macrophages and can be further induced by stimulation with bacterial-derived pro-inflammatory stimuli LPS, immune regulatory T-helper type 2 cytokines IL-4 and IL-13, and HIF-1 following reduced oxygen tension. ALOX15 is absent in human monocyte-derived macrophages and requires stimulation by IL-4 or IL-13 for protein induction. Macrophages exposed to apoptotic cells (ACs) and IL-4 or IL-13 display pro-resolving properties exemplified by heightened ALOX15 expression. Question marks indicate uncertain signaling pathways.

In contrast to ALOX15, which is absent in unstimulated human monocyte-derived macrophages, ALOX15B is constitutively expressed at both messenger RNA (mRNA) and protein levels, and its expression can be further enhanced by bacterial-derived pro-inflammatory stimulus LPS and immune regulatory T-helper type 2 cytokines IL-4 and IL-13 (**Figure 2**) (Wuest et al., 2012; Snodgrass et al., 2018).

Gene and protein expression levels as well as enzymatic activity of ALOX15B were also increased in primary human macrophages incubated under hypoxia and following treatment with dimethyloxalylglycine, which mimics low oxygen tension by stabilizing the transcription factor hypoxia-inducible factor 1α (HIF-1α) (Rydberg et al., 2004; Hultén et al., 2010). Neither dimethyloxalylglycine nor hypoxia increased expression of ALOX15. Furthermore, knockdown of HIF-1α in hypoxic macrophages reduced production of the 15-lipoxygenase-mediated AA metabolite 15-HETE, further implicating a role for HIF-1α in the hypoxic induction of ALOX15B. More detailed studies investigating the regulation of *ALOX15B* gene expression were performed in normal human prostate (HNP) epithelial cells (Tang et al., 2004; Bhatia et al., 2005). Analysis revealed that the *ALOX15B* promoter is not TATA box-enriched, consistent with the constitutive expression *in vivo*, and contains several Sp1 sites critical for regulating gene expression. Subsequent experiments identified Sp1 and Sp3 as major GC-binding *trans* factors regulating *ALOX15B* gene expression. Sp1 activated while Sp3 inhibited *ALOX15B* promoter activity, and endogenous *ALOX15B* expression in HNP cells established Sp1 and Sp3 as biologically relevant and essential regulators of the *ALOX15B* gene (Tang et al., 2004).

Several splice variants of ALOX15B were identified in HNP epithelial cells and prostate cancer (PCa) cells (Bhatia et al., 2003; Bhatia et al., 2005; Tang et al., 2007). All identified variants had spliced out key segments of the protein important for its catalytic activity, leaving the enzyme with little to no AA-metabolizing activity. Unlike the full-length protein, the alternatively spliced isoforms were also found to be largely excluded from the nucleus. In gain-of-function experiments, splice variant-expressing cells displayed similar biological activities to the full-length protein with respect to inhibiting cell-cycle progression and tumor development and inducing cell senescence. These results suggest that the tumor-suppressive functions of ALOX15B and its splice variants do not necessarily depend on AA-metabolizing activity and nuclear localization and support a biological function for ALOX15B independent of PUFA metabolism.

In human monocyte-derived macrophages, ALOX15 expression is strictly cytokine-dependent and is strongly up-regulated following stimulation with IL-4 or IL-13 (Conrad et al., 1992; Czimmerer et al., 2012; Gundra et al., 2014; Ackermann et al., 2017). Stimulation with other cytokines including IL-1β, IL-6, TNF-α, TGF-β, and IFNγ, by agonists of TLR3, TLR4, TLR7, TLR8, TLR9, as well as hypoxia, did not induce expression of ALOX15 mRNA or protein (Rapp et al., 1983; Belkner et al., 1993; Wuest et al., 2012). Maximal protein induction is reached after incubation periods longer than 48 h, suggesting that *ALOX15* does not belong to the immediate early genes of the IL-4 response (Wuest et al., 2012; Werz et al., 2018). *In vitro* experiments posit that activation of the transcription factor signal transducer and activator of transcription (STAT)-6 by IL-4 or IL-13 is indispensable for induction of *ALOX15* gene transcription in monocyte-derived macrophages (Heydeck et al., 1998; Han et al., 2014). Although both IL-4 and IL-13 signal through receptor systems containing the IL-4Rα component to induce *ALOX15* in a STAT6-dependent manner, their intracellular signaling mechanisms are distinct. IL-4 utilizes the IL-4Rα/Jak1 cascade to activate STAT6 and STAT3 whereas IL-13 utilizes both IL-4Rα/Jak2 and IL-13Rα1/Tyk2 to activate STAT6, STAT3, and STAT1 (Bhattacharjee et al., 2013). Histone modifications and chromatin remodeling also play critical roles in *ALOX15* gene expression. In human lung epithelial carcinoma A549 cells, IL-4 activated the histone acetyltransferase activity of the cAMP response element binding protein (CREB)-binding protein (CBP)/p300, which is responsible for acetylation of nuclear histones and STAT6 (Shankaranarayanan et al., 2001). Inhibition of its acetyltransferase activity abrogated acetylation of both histones and STAT6 and strongly suppressed transcriptional activation of *ALOX15*. In the Hodgkin lymphoma cell line L1236, which constitutively express *ALOX15*, abolishing histone methyltransferase SMYD3 reduced *ALOX15* expression by reducing di-/trimethylation of histone 3 lysine 4 (H3-K4), attenuated occupancy of STAT6, and diminishing histone H3 acetylation at the *ALOX15* promoter. In contrast to L1236 cells, inhibiting JmjC-domain-containing H3-K4 tri-demethylase lysine demethylase 5C (KDM5C) in the Hodgkin lymphoma cell line L428, which does not express *ALOX15*, upregulated *ALOX15* expression through inducing H3-K4 trimethylation, histone acetylation, and STAT6 recruitment at the *ALOX15* promoter (Liu et al., 2012). In A549 cells but not human peripheral monocytes, IL-4 stimulation induced H3K27me2/3-specific demethylase (UTX)-mediated H3K27me3 demethylation at the *ALOX15* promoter, triggering mRNA transcription and protein expression (Han et al., 2014). Further investigations showed that IL-13-mediated *ALOX15* gene expression in human primary monocytes involves ERK1/2-dependent signaling cascades that induce transcription factor early growth response-1 (Egr-1) nuclear accumulation and CREB serine phosphorylation and subsequent DNA binding to their cognate sequences within the *ALOX15* promoter (Bhattacharjee et al., 2010). In primary human monocyte-derived macrophages, IL-4-induced *ALOX15* expression was attenuated by AMP-activated protein kinase (AMPK) activation. AMPK activation inhibited IL-4-evoked STAT3 but not STAT6 activation. In addition, AMPK activation prevented IL-4-induced association of STAT6 and Lys-9 acetylation of histone H3 at the *ALOX15* gene promoter (Namgaladze et al., 2015).

Currently, the only known mode of ALOX15 induction in monocytes and macrophages is through stimulation with classical Th2 cytokines IL-4 or IL-13 (Kuhn et al., 2016). However, the discrepancy between concentrations of IL-4 and IL-13 required to induce ALOX15 in cell culture models (typically 1–50 ng/ml) with those measured in human plasma (low pg/ml range) questions the biological relevance of IL-4- and IL-13-induced upregulation of ALOX15 expression (Kuhn et al., 2016). Considerations about the dispensable nature of IL-4- and IL-13-induced ALOX15 expression *in vivo* stem from early investigations comparing ALOX15 expression in human monocyte-derived macrophages matured *in vitro* with *in vivo*-matured mouse peritoneal macrophages. In 1996, it was shown that mouse peritoneal macrophages possess considerable Alox15 activity and protein in the absence of exogenous IL-4 and when harvested from IL-4-deficient mice (Cornicelli et al., 1996). However, the subsequent discovery that IL-13, which uses shared receptor subunits to activate common pathways as IL-4, mimics the effect of IL-4 on ALOX15 expression in monocytes and macrophages (Nassar et al., 1994; Heydeck et al., 1998; Chaitidis et al., 2005) likely account for the lack of reduced Alox15 expression in macrophages isolated from IL-4-deficient mice. Follow-up experiments investigating the dispensable nature of lymphocyte-derived IL-4 and IL-13 in Alox15 expression *in vivo* assessed Alox15 levels in peritoneal macrophages harvested from recombinase activator gene (RAG)-2 knockout mice deficient in Th2 cytokine-producing mature lymphocytes (Sendobry et al., 1998). In contrast to the researchers’ expectations, Alox15 levels in macrophages isolated from knockout mice were not decreased. However, in the 20 years since this publication, many additional cell types in addition to mature T lymphocytes have been shown to produce IL-4 or IL-13 including NK T cells, basophils, mast cells, eosinophils, group 2 innate lymphoid cells (ILC2 cells), and multipotent progenitors type 2 cells (Molofsky et al., 2013; Paul, 2015; Yamanishi and Karasuyama, 2016). More recent studies have shown that the sensing of IL-4 or IL-13 together with the recognition and integration of ACs through members of the TAM (Tyro3, Axl, and Mer) receptors, which are expressed on professional phagocytes and contribute to AC clearance, enhances anti-inflammatory and tissue repair gene expression including *Alox15* (Bosurgi et al., 2017). ACs are generated not only during inflammation but also under normal physiological conditions (Poon et al., 2014), resulting in more than 10^11^ ACs cleared in the normal adult mammal on a daily basis (Henson, 2017). Considering the ubiquity of ACs *in vivo* and their demonstrated ability to enhance IL-4- and IL-13-induced *Alox15* expression in murine macrophages, investigating how macrophages integrate concurrent recognition of biological factors and cytokine receptor signals may reveal novel mechanisms coordinating ALOX15 expression *in vivo*.

## Biological Function of ALOX15 and ALOX15B in Human Macrophages

### Production of SPMs

Initiation and resolution of inflammation are finely regulated by chemical messengers including lipid mediators (Spite et al., 2014). Whereas AA-derived prostaglandins and leukotrienes formed by the cyclooxygenase and arachidonate 5-lipoxygenase (ALOX5) pathways initiate acute inflammation, the process of terminating inflammation and promoting resolution are coordinated by a group of temporally produced lipids called SPMs. The SPM superfamily consist of LXs synthesized from AA, E-series Rvs from EPA, and DHA-derived D-series Rvs, protectins, and maresins (Spite et al., 2014). While enzyme activity assays show both ALOX15 and ALOX15B synthesize SPM precursor lipids including 15-HETE used for LX synthesis and 17-HDHA used for the synthesis of Rvs (Kutzner et al., 2017), biological data in macrophages posit that only ALOX15 contribute to this process. Stimulation of ALOX15B-expressing human monocyte-derived macrophages with IL-4 increased protein expression of ALOX15 and cellular levels of 15-LOX-synthesized SPM precursor 15-HETE and monohydroxy LA metabolite 13-HODE (Snodgrass et al., 2018). In these cells, only knockdown of ALOX15, but not ALOX15B, decreased the lipid metabolites to basal levels, suggesting that only ALOX15 contributes to SPM precursor generation. Moreover, LPS- and interferon-γ-polarized classically activated macrophages expressing ALOX5, 5-lipoxygenase-activating protein (FLAP), but not ALOX15 challenged with pathogenic *Escherichia coli* produced large amounts of prostaglandin E_2_ and leukotriene B_4_ but no SPMs or 15-LOX-derived SPM precursors (Werz et al., 2018; Werner et al., 2019). In IL-4-polarized alternatively activated macrophages, which strongly express ALOX15 and contain low levels of FLAP, incubation with *E. coli* produced 15-LOX-derived SPM precursors (15-HETE, 17-HDHA, and 15-HEPA) and SPMs (RvD2, RvD5, LXs, and maresin). Although ALOX15B protein is constitutively expressed in human macrophages (Wuest et al., 2012; Snodgrass et al., 2018), only ALOX15 protein appears to contribute to the production of SPMs and its precursors.

### ACs and Efferocytosis

ALOX15 is highly expressed in IL-4- and IL-13-induced alternatively activated macrophages *in vitro* and in macrophages participating in the resolution of inflammation *in vivo* (Schif-Zuck et al., 2011; Czimmerer et al., 2012; Uderhardt et al., 2012; Gundra et al., 2014; Ackermann et al., 2017; Snodgrass et al., 2018; Werz et al., 2018; Yang et al., 2019). Transcriptomic analysis of resolution-phase macrophages showed them to be enriched in genes involved in antigen processing and presentation as well as genes involved in dampening leukocyte trafficking, wound repair, and efferocytosis (Stables et al., 2011). Subsequent analysis of Alox15-expressing resolution-phase macrophages isolated from models of resolving peritonitis and acute *N*-acetyl-p-aminophenol-induced liver injury indicated a heightened efferocytic capacity (Schif-Zuck et al., 2011; Uderhardt et al., 2012; Yang et al., 2019). However, rather than facilitating the uptake of ACs, Alox15 expression appears to be a consequence of AC engulfment, as post-efferocytotic “satiated” Alox15-expressing macrophages were shown to display reduced responsiveness to TLR ligands and low phagocytic potential, and were prone to migrate to lymphoid organs (Schif-Zuck et al., 2011). An independent study of peritonitis also excluded an intrinsic role for Alox15 during the ingestion of ACs as resolution-phase macrophages from both wild-type and Alox15 knockout mice ingested equally high amounts of ACs (Uderhardt et al., 2012). Although likely not required for the direct uptake of ACs, the enzyme appears to be a crucial factor orchestrating clearance of ACs as resident peritoneal macrophages utilize Alox15 to generate specific oxidation products of PE, which block uptake of ACs from recruited inflammatory monocytes. This selective phagocytosis prevents efferocytosis by inflammatory monocytes and subsequent antigen presentation of AC-derived antigens, thus maintaining self-tolerance.

Recent studies from animal models suggest the reprogramming of AC-engulfing macrophages to an Alox15-expressing, pro-resolving, and tissue repair phenotype that involves Axl and Mer tyrosine kinase (MerTK) TAM receptors (Bosurgi et al., 2017; Lumbroso et al., 2018). Mice infected with the helminth *Nippostrongylus brasiliensis* develop substantial lung tissue damage followed by a rapid IL-4 and IL-13 response, which is critical for resolution of inflammation and tissue repair. Lung-resident macrophages isolated 7 days post *N. brasiliensis*-infection showed increased expression of *Alox15* as well as anti-inflammatory and tissue repair genes that were substantially reduced in mice with macrophage-specific ablation of the AC sensors Axl and MerTK (Bosurgi et al., 2017). The expression of IL-4-induced genes that were independent of AC sensing was not impaired in macrophages lacking Axl and MerTK. To gain further insight into macrophage reprogramming by TAM receptor signaling, Lumbroso et al. utilized macrophages deficient in the bridging molecule Pros1, which binds PS on ACs to allow TAM receptor engagement, in a zymosan-induced peritonitis mouse model (Lumbroso et al., 2018). Pros1-deficient macrophages collected from peritoneal exudates 66 h post zymosan treatment engulfed fewer apoptotic polymorphonuclear cells (PMNs) compared to control cells. Moreover, Pros1-deficient peritoneal macrophages displayed reduced reprogramming following apoptotic neutrophil engulfment as indicated by increased secretion of pro-inflammatory mediators and decreased levels of anti-inflammatory cytokines following exposure to LPS. Pros1-deficient peritoneal macrophages also expressed reduced levels of Alox15 protein and produced 25% less RvD1 compared to control cells. Collectively, these studies indicate the significance of the coordination of IL-4 or IL-13 with AC sensing in macrophages in inducing ALOX15 and the anti-inflammatory and tissue repair phenotype. Although the molecular signaling mechanisms that integrate IL-4, IL-13, and AC sensing to reprogram macrophages remain unknown, it is interesting to consider how constitutively expressed ALOX15B might play a role in this process and whether it displays any intrinsic or coordinating role in efferocytosis.

### Atherosclerosis

The role of ALOX15 and ALOX15B in atherogenesis is complex due in part to the fact that atherosclerosis is a multifactorial disease entailing a complex interplay of modified lipoproteins, monocyte-derived macrophages, T cells, and the arterial wall. Since macrophages play a central role in atherogenesis, understanding the role of macrophage-specific 15-LOXs in lipid handling and foam cell formation has been of great interest. Oxidative modification of LDL particles in the artery wall leads to the formation of atherogenic oxidized LDL (oxLDL). oxLDL can be recognized and taken up by macrophages where the accumulation of cholesterol converts them into foam cells, which initiate the development of atherosclerotic lesions. Although the precise mechanisms that generate oxLDL *in vivo* are still only partially understood, both ALOX15 and ALOX15B may contribute to the formation of atherogenic oxLDL. Reports have shown that rabbit, mouse, porcine, and human ALOX15 directly oxidizes LDL particles and contributes to foam cell formation (Belkner et al., 1993; Kühn et al., 1994; Kühn and Chan, 1997; Belkner et al., 1998; Heydeck et al., 2001). Although these results support the notion that ALOX15 can directly contribute to atherosclerosis *via* LDL oxidation, subsequent studies performed in transgenic animal models reported both pro-atherogenic and anti-atherogenic roles for ALOX15 (Shen et al., 1996; Cyrus et al., 1999; Harats et al., 2000; Merched et al., 2008). Early studies that analyzed human atherosclerotic plaques reported increased levels of enzymatically derived 13(S)-HODE compared to the non-enzymatically-derived 13(R)-HODE, suggesting the presence of ALOX15 and its contribution to the formation of atherosclerotic lesions (Kühn et al., 1994; Folcik et al., 1995; Kühn et al., 1997). However, with the discovery of a second 15-lipoxygenase, ALOX15B, a more thorough analysis and comparison of the role of the 15-LOXs in atherosclerosis became warranted. It is now well established that human atherosclerotic plaques contain large amounts of 15-lipoxygenase-derived lipid metabolites of AA, EPA, and DHA; however, the respective contribution of ALOX15 and ALOX15B is unclear (Fredman et al., 2016). Using a collection of human carotid plaque tissue, Gertow et al. reported that *ALOX15B* was highly expressed in the carotid lesions while immunohistochemical analysis showed abundant ALOX15B protein expression in macrophage-rich lesion areas (Gertow et al., 2011). In contrast to ALOX15B, *ALOX15* expression was not detected in carotid lesions. A second study using human carotid plaques from patients with high-grade symptomatic carotid artery stenosis also reported high ALOX15B but not ALOX15 protein levels in carotid lesions (Hultén et al., 2010). Immunohistochemistry analysis showed that ALOX15B expression in carotid endarterectomies correlated with the expression of HIF-1α. Moreover, *ALOX15B* gene expression in CD14+ macrophages isolated from the human carotid endarterectomies was 500 times higher than *ALOX15*. To investigate the role of ALOX15B in promoting the development of atherosclerosis *in vivo*, researchers used lentiviral shRNA silencing and bone marrow transplantation to knock down Alox15b in LDL-receptor-deficient mice (Magnusson et al., 2012). Immunohistochemical analysis indicated mice that received Alox15b knockdown bone marrow had reduced atherosclerotic lesions in both whole aorta and aortic root compared to nonsilencing control mice. In summary, although *in vitro* data support a role for ALOX15 and ALOX15B in atherogenesis, data from *in vivo* animal experiments and from human carotid plaque tissue implicate a role for ALOX15B rather than ALOX15.

### Cellular Lipid Homeostasis

Various studies have reported roles for the 15-LOX enzymes in regulating macrophage lipid homeostasis (Belkner et al., 2005; Weibel et al., 2009; Rong et al., 2012; Snodgrass et al., 2018). Initial experiments found that overexpressing porcine Alox15 in J774 murine macrophages protected cells from intracellular lipid deposition following incubation with acetylated LDL (Belkner et al., 2005). Further analysis discovered that porcine Alox15-overexpressing macrophages accumulated less intracellular CEs due to increased CE catabolism. Administration of Alox15-mediated metabolites of AA and LA, 12-HETE and 13-HODE, failed to reduce intracellular CE accumulation in control cells, suggesting a mechanism mediated *via* metabolites of free and/or esterified hydroperoxy lipids formed from Alox15-catalyzed fatty acid oxygenation. In a similar experiment, overexpression of human ALOX15 in RAW 267.4 murine macrophages led to increased intracellular CE hydrolysis, elevated ABCA1 protein levels, and enhanced cholesterol efflux, suggesting that the CEs produced in ALOX15-expressing cells are readily mobilized for ABCA1-mediated cholesterol efflux (Weibel et al., 2009). The increased intracellular CE hydrolysis observed in ALOX15-expressing macrophages was not attributable to increased neutral cholesterol ester hydrolase activity, suggesting that the CEs in ALOX15-expressing cells are a better substrate for neutral cholesterol ester hydrolase compared to CEs in control cells. Although consistent with previous experiments showing that ALOX15 oxygenates intracellular CE, levels of oxCE in the ALOX15-overexpressing and control cells were not measured in these experiments. In an animal model of atherosclerosis, Alox15/Ldl receptor double knockout mice fed a PUFA-enriched diet had reduced plasma cholesterol and triglyceride levels, liver lipid levels, and aortic atherosclerosis compared to Ldl receptor knockout mice (Rong et al., 2012). Hepatic gene expression revealed that double knockout mice had reduced levels of fatty acid and triglyceride synthesis-related genes sterol regulatory element binding protein-1c (*Srebp-1c*), fatty acid synthase (*Fas*), acetyl-CoA carboxylase-1 (*Acc-1*), stearoyl-CoA desaturase-1 (*Scd-1*), as well as cholesterol synthetic regulatory genes *Srebp-2* and 3-hydroxy-3-methylglutaryl-CoA (*Hmg-CoA*) synthase and reductase compared with single knockout controls. Considering that ALOX15 is expressed in macrophages, not hepatocytes, the authors concluded that macrophage ALOX15 expression altered secretory products that affected hepatic lipid synthesis. With respect to lipid and sterol homeostasis in macrophages, we recently reported that suppressing ALOX15B and, to a lesser extent, ALOX15 in human primary monocyte-derived macrophages impaired SREBP-2 signaling by inhibiting SREBP-2 processing into mature transcription factor and reduced SREBP-2 binding to sterol regulatory elements and subsequent target gene expression (Snodgrass et al., 2018). In IL-4 stimulated human macrophages, which express both ALOX15 and ALOX15B proteins, silencing ALOX15B but not ALOX15 reduced cellular cholesterol and the cholesterol intermediates desmosterol, lanosterol, 24,25-dihydrolanosterol, and lathosterol as well as oxysterol 27-hydroxycholesterol. In addition to reduced expression of SREBP-2 target genes, knockdown of ALOX15B increased expression of liver X receptor (LXR) target genes *ABCG1*, *ABCA1*, and *MYLIP* following stimulation with IL-4. In agreement with previous reports, attempts to rescue alterations in SREBP-2 target gene expression in 15-LOX-suppressed macrophages with exogenous AA-, LA-, or DHA-derived 15-LOX metabolites failed. Collectively, our results are in agreement with previous reports suggesting a role for 15-lipoxygenases in regulating lipid and sterol homeostasis in macrophages through a currently undefined mechanism.

### Potential Functions of Macrophage ALOX15B

In contrast to macrophage ALOX15, which is strongly implicated in inflammation resolution and orchestrating efferocytosis, no compelling data currently exist to suggest a role for ALOX15B in either of these processes. Whereas most of the *in vivo* biological functions of ALOX15 have been elucidated using knockout mice, Alox15b-deficient mice are not commercially available. Likewise, many potent small-molecule inhibitors targeting ALOX15 have been reported (Rai et al., 2013; Sadeghian and Jabbari, 2016) and are commercially available, including PD146176 and ML351; however, inhibitors exhibiting potent and selective inhibition of ALOX15B do not currently exist. Therefore, the limited collective knowledge of the biological functions of ALOX15B is derived primarily from *in vitro* experimentation through manipulating its expression in various cell culture models. While the function of ALOX15B in macrophages is rather unclear, its role in non-myeloid cells including human epithelial cells of the prostate, skin, esophagus, and cornea has been more thoroughly investigated (Tang et al., 2002; Schweiger et al., 2007; Tang et al., 2007; Suraneni et al., 2014). In epithelial cells, ALOX15B functions as a regulator of cell senescence (Schweiger et al., 2007; Tang et al., 2007; Suraneni et al., 2010). Research in human prostate cells has shown that ALOX15B expression is down-regulated or lost in the precursor lesion HGPIN (high-grade prostate intraepithelial neoplasia) as well as in >70% of prostate cancers (Shappell et al., 1999; Jack et al., 2000; Tang et al., 2002; Tang et al., 2007; Suraneni et al., 2014). In nearly all immortalized prostate epithelial and PCa cell lines, the expression of ALOX15B is undetectable. Furthermore, restoring its expression in PCa cells inhibits proliferation, induces senescence-like phenotypes, and abrogates tumor regeneration in xenograft models (Suraneni et al., 2014). Although mechanisms by which ALOX15B functions as a negative cell cycle regulator are not well characterized, it likely possesses multiple biological functions as gain-of-function experiments using ALOX15B splice variants lacking AA-metabolizing activity produced identical biological activities compared with the full length protein. These activities include the inhibition of cell-cycle progression and proliferation, induction of a senescence-like phenotype, and inhibition of tumor development *in vivo*. To gain a better understanding of how ALOX15B and its splice variants contribute to cellular homeostasis in health and disease, additional research is needed to elucidate the cellular functions of ALOX15B.

## Conclusion

Since the discovery of ALOX15 in 1975 (Schewe et al., 1975), an understanding of its function and physiological significance has been extensively pursued. It is now well established that ALOX15 is an IL-4- and IL-13-inducible enzyme in human monocyte-derived macrophages that catalyzes the oxygenation of free and esterified PUFAs. Through its lipid-metabolizing activity, macrophage ALOX15 plays a central role in generating SPMs to resolve acute inflammation and to produce oxPLs to orchestrate the nonimmunogenic removal of ACs. In 1997, a second AA 15-lipoxygenating enzyme was discovered in human skin and, based on LOX nomenclature, was given the name ALOX15B (Brash et al., 1997). Since then, comparative analyses of ALOX15 and ALOX15B have shown that the former enzyme exhibits dual reaction specificity with several polyenoic fatty acids while the latter exhibits singular reaction specificity. In contrast to ALOX15, which is absent in unstimulated human monocyte-derived macrophages, ALOX15B is constitutively expressed but does not appear to play a major role in inflammation resolution or orchestrating efferocytosis. Although several correlations between ALOX15B expression and biological activities in non-macrophage cells have been reported, the function of ALOX15B in human macrophages remains elusive.

## Author Contributions

This manuscript was conceptualized by RS and BB, written by RS, and edited by RS and BB.

## Funding

This work was supported by the Deutsche Forschungsgemeinschaft (SFB 1039, TPB04 and GRK 2336, TP06).

## Conflict of Interest Statement

The authors declare that the research was conducted in the absence of any commercial or financial relationships that could be construed as a potential conflict of interest.

## Abbreviations

AA, arachidonic acid; AC, apoptotic cell; ALOX5, arachidonate 5-lipoxygenase; ALOX15, arachidonate 15-lipoxygenase; ALOX15B, arachidonate 15-lipoxygenase type B; AMPK, AMP-activated protein kinase; CE, cholesteryl ester; DHA, docosahexaenoic acid; EPA, eicosapentaenoic acid; HEPA, hydroxyeicosapentaenoic acid; HETE, hydroxyeicosatetraenoic acid; HDHA, hydroxydocosahexaenoic acid; HIF-1α, hypoxia-inducible factor 1α; HNP, normal human prostate; HODE, hydroxyoctadecadienoic acid; LA; linoleic acid; LDL, low-density lipoprotein; LOX, lipoxygenase; LPS, lipopolysaccharide; LX, lipoxin; oxCE, oxidized CE; oxLDL, oxidized LDL; oxPL, oxidized phospholipid; PC, phosphatidylcholine; PCa, prostate cancer; PE, phosphatidylethanolamine; PEBP1, phosphtidylethanolamine-binding protein 1; PL, phospholipid; PUFA, polyunsaturated fatty acid; Rv, resolvin; SPM, specialized pro-resolving mediator; STAT, signal transducer and activator of transcription.
